# Right-side versus left-side hemihepatectomy for the treatment of Bismuth type IV perihilar cholangiocarcinoma: a comparative study

**DOI:** 10.3389/fonc.2025.1663334

**Published:** 2025-11-26

**Authors:** Wencong Ma, Mingtai Hu, Zhihua Xie, Jianyang Ao, Peining Yan, Yao Huang, Jinghan Wang, Xiaoqing Jiang

**Affiliations:** 1 Institute of Hepatobiliary and Pancreatic Surgery, Department of Hepatobiliary and Pancreatic Surgery, Shanghai East Hospital, School of Medicine, Tongji University, Shanghai, China; 2 Department of Biliary Tract Surgery I, The Third Hospital of Naval Medical University, Shanghai, China

**Keywords:** cholangiocarcinoma, perihilar cholangiocarcinoma, locally advanced cholangiocarcinoma, Bismuth type IV, hemihepatectomy

## Abstract

**Objective:**

Radical surgical resection is the only potentially curative treatment for perihilar cholangiocarcinoma (PHC) patients. However, data on left-sided hemihepatectomy (LH) and right-sided hemihepatectomy (RH) outcomes for Bismuth-Corlette type IV PHC are scarce and controversial. This study aimed to explore surgical and long-term outcomes of LH and RH in these patients.

**Methods:**

Medical records of Bismuth type IV PHC patients who had liver resection from 2009 to 2018 were retrospectively analyzed. Surgical results and long-term survival were the primary outcomes, compared via one-to-one propensity score matching (PSM).

**Results:**

218 Bismuth type IV PHC patients (146 LH, 72 RH) were analyzed. The RH group had a higher proportion of preoperative biliary drainage (p = 0.02) and more frequent portal vein embolization (p < 0.0001). R0 resection rate was 90.37% (197/218) with no significant LH-RH difference. Post-operative severe complication (grades 3-5) and 90-day mortality rates were comparable. Overall survival was similar (overall cohort: p=0.21; matched cohort: p=0.54). But in the overall cohort, R0-resected RH patients had marginally better survival (p = 0.064). Prognostic factors included carbohydrate antigen 19-9 (CA19-9), age, tumor vascular invasion, and severe post-operative complications.

**Conclusions:**

The postoperative morbidity and mortality rate was comparable between LH and RH for Bismuth type IV PHC. Although RH showed a favorable survival from the Kaplan-Meier survival curve, no significant difference was observed in overall survival after LH versus RH for the overall cohort and the matched cohort after PSM.

## Introduction

Perihilar cholangiocarcinoma (PHC) is a rare, complex, and intractable malignancy with a poor prognosis (1, 2). Because of the complex anatomical structure and diversity of anatomic variation of the liver hilum (3), radical surgical resection of the PHC remains a big challenge for hepatobiliary surgeons. But aggressive surgical treatment, including hemihepatectomy to the caudate lobe, extrahepatic bile duct resection, and radical lymphadenectomy for PHC, may offer the only chance for a cure and substantial overall survival (OS) benefits, especially for the Bismuth-Corlette type III and IV PHC (4–6). However, the choice of left-sided hemihepatectomy (LH) or right-sided hemihepatectomy (RH) remains controversial (6–8), especially for Bismuth type IV perihilar cholangiocarcinoma.

Generally, the determination of whether patients undergo a right-sided or left-sided liver resection is based on the predominant, anatomic location of the tumor, vascular involvement, and future liver remnant (FLR) (9). A number of other factors also affect the surgical choice. The longer extrahepatic section of the left hepatic duct, compared to the right hepatic duct, results in a longer distance from the hepatic bifurcation to the surgical margins in the left liver than in the right liver, which was favorable for achieving a histologically negative margin (R0) after RH. More importantly, the right hepatic artery generally passes behind the common bile duct, close to the surface of the ductal confluence, which was susceptible to the involvement by tumor. While the left hepatic artery enters into the liver from the umbilical fissure, located well away from the common bile duct, and was rarely involved by the tumor. Therefore, the RH, including the resection of the right hepatic artery, may have an anatomic advantage for radicality (5, 10, 11). Moreover, owing to the anatomic characteristics of the hepatic hilus, the procedure of LH is more complex and requires greater surgical skill than RH (12).

However, the future liver remnant after RH was smaller than the corresponding LH, which was consequently associated with greater postoperative liver dysfunction, even postoperative mortality (8, 13). Some studies have suggested that as the increasing ability to perform potentially curative LH for PHC, the safety and survival of LH was comparable to RH (7, 13). Moreover, the RH requires a more optimized plan of the future liver remnant with preoperative biliary drainage (PTBD) and/or portal vein embolization (PVE), and the patients have to bear the additional time, expense, and associated risk (14).

To date, past studies have produced conflicting conclusions, and available data was limited. During the past 20 years, our department adopted a policy of aggressive surgical resection for PHC, even those with vascular involvement (15, 16). Therefore, the aim of this study was to compare the clinicopathologic outcomes and postoperative morbidity and mortality rates between the left-sided and right-sided hepatectomy for the Bismuth type IV PHC.

## Methods

### Patients

**Figure 1:**
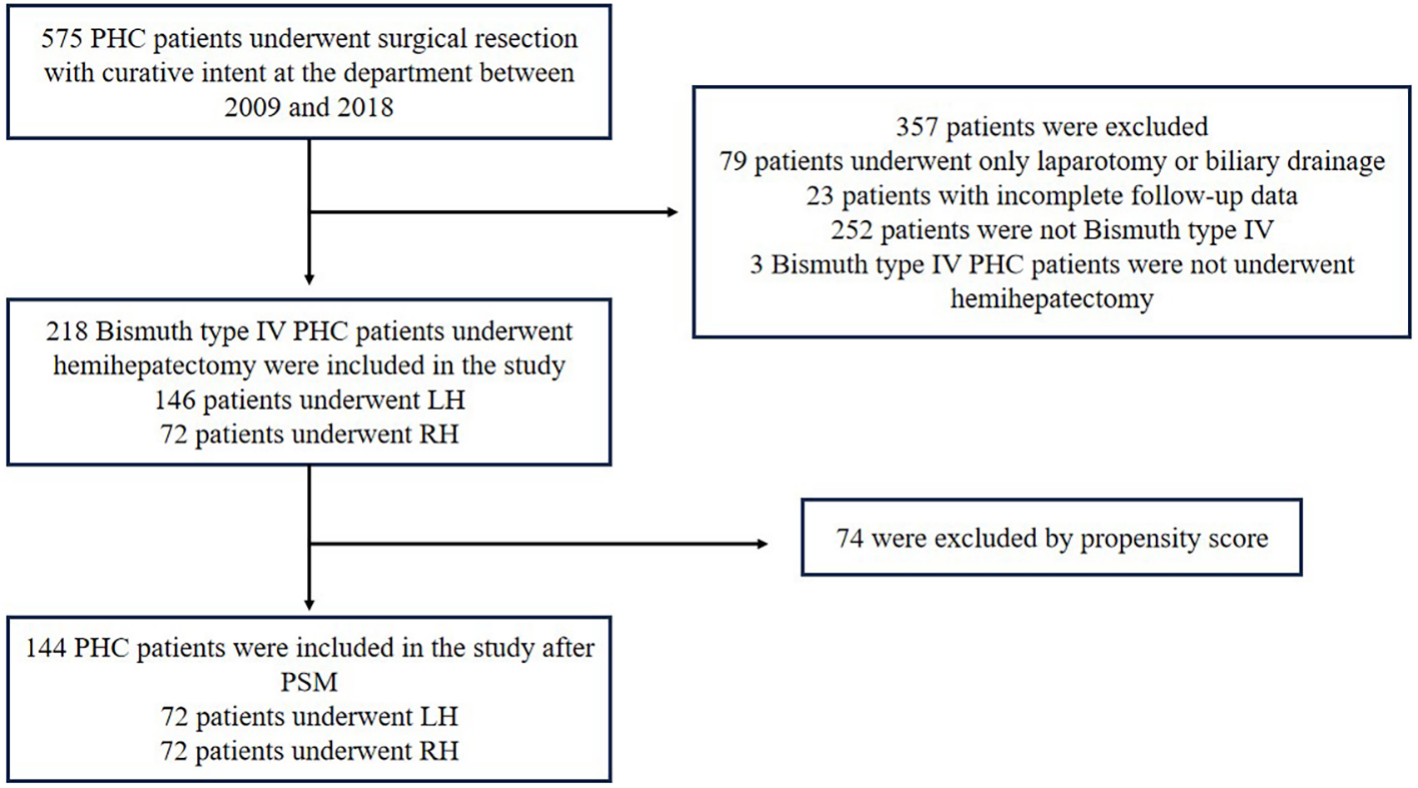
Flow chart to select Bismuth type IV PHC patients for the study.

From January 2009 to December 2018, a total of 575 patients with PHC (Bismuth type I-IV) underwent surgical resection with curative intent. The baseline characteristics of these 575 patients have been described previously in a prior study (15). Of these patients, 218 Bismuth type IV PHC patients who underwent RH or LH with extrahepatic bile duct resection were eligible for this study (**Figure 1**). The study was approved by the Institutional Ethics Committee of the hospital.

### Preoperative workup and management

Contrast-enhanced multidetector computed tomography (CT) and magnetic resonance (MR) cholangiopancreatography were routinely employed to evaluate the longitudinal and vertical extension of the tumor and then the resectability and hilar vascular structures. Positron emission tomography (PET)-CT was performed if necessary to rule out potential distant metastases. Endoscopic nasobiliary drainage (ENBD), endoscopic retrograde biliary drainage (ERBD), and percutaneous transhepatic biliary drainage (PTBD) were aggressively conducted to decrease the total serum bilirubin (TBIL) level. The drainage strategy for the FLR was inclined to use PTBD. If the FLR was expected to be less than 40% of total liver volume, portal vein embolization (PVE) was undertaken to induce the hypertrophy of the future remnant liver (17).

### Surgical procedures and follow-up

The decision regarding whether a left-sided or right-sided hepatectomy was performed was made on the basis of the predominant tumor site as well as the future remnant liver volume. In general, LH was mainly undertaken for Bismuth type IIIb and most of the Bismuth type IV tumors, while RH was for Bismuth type IIIa and a part of the Bismuth type IV tumors. En bloc resection of the caudate lobe, extrahepatic bile duct, and lymph node dissection in the hepatoduodenal ligament was performed in almost all patients undergoing hepatectomy.

Each patient was followed up regularly, and the follow-up visits comprised a physical exam, laboratory tests including tumor markers, and radiologic cross-sectional imaging (CT or MRI scan). Overall survival (OS) and 90-day mortality were the endpoints of this study.

### Tumor definition and classification of complications

The Bismuth–Corlette classification was used to categorize the type of PHC by various imaging scan methods before surgery (18). Histopathological characteristics and staging were classified according to the Union for International Cancer Control (UICC) TNM 8th edition staging criteria for HCCA (19). Curative (R0) resection was defined as no residual cancer at all surgical margins, such as the hepatic ductal margin, distal ductal margin, and radial margin. A microscopic positive resection margin was defined as R1 resection, while macroscopic evidence of residual tumor was defined as R2 resection. Postoperative complications were graded retrospectively according to the Clavien-Dindo classification of surgical complications (20). The severe complications were defined as those of Clavien-Dindo grades III, IV, and V. Postoperative mortality was defined as all deaths during the hospital stay or within 90 days after surgery.

### Chemotherapy and radiotherapy

The postoperative adjuvant treatment protocol has been described in detail in the previous article (15). In brief, patients with negative resection margin (R0) were attempted to receive S1 chemotherapy only. Patients with R1 resection received S1 plus platinum-based drugs. Chemotherapy or radiotherapy was administered for patients with R2 resection depending on the patient’s wishes.

### Propensity score matching

The propensity score matching (PSM) method was utilized to control selection bias and to compare surgical and survival outcomes in matched groups of patients who underwent LH or RH. The propensity score (PS) was estimated using a logistic regression of the treatment on the covariates, considering age, gender, total bilirubin at diagnosis, total bilirubin at operation, carbohydrate antigen 19-9 (CA19-9) levels at operation, preoperative biliary drainage, and PVE. Patients were matched in a 1:1 ratio through nearest neighbor matching without replacement, based on the estimated propensity score.

### Statistical analysis

Continuous variables following a normal distribution were described by mean and standard deviation or median and interquartile range in case of a non-normal distribution. Comparison of continuous variables between groups was performed using the t-test for means and the Mann-Whitney test for medians. Categorical variables were compared using chi-square or Fisher’s exact tests, as appropriate. Postoperative patient survival was calculated using the Kaplan-Meier method. Comparison of patient survival between groups was performed using the log-rank test. To identify predictors of survival among the patients who underwent LH or RH, univariate and multivariate analyses were performed according to the Cox proportional hazards regression model. The factors found to be significant in the univariate analysis (P ≤ 0.1) were subjected to multivariate analysis. The optimal cut-off values of continuous variables (age, total bilirubin at diagnosis, total bilirubin at operation, and CA19–9 levels) for differentiation between the groups were identified by X-tile (Yale University, version 3.6.1). All data were expressed as mean plus standard deviation or as median and range when appropriate. Significance was defined as p<0.05. Statistical analyses were performed using R-4.4.1. Survival curves were displayed using R-4.4.1.

## Results

### Preoperative characteristics

The demographics and characteristics of 218 Bismuth type IV PHC patients who underwent hemihepatectomy (Major) or extended hemihepatectomy (Extended) with curative intent are summarized in **Table 1**. These included 141 males (64.68%) and 77 females (35.32%) with a median age of 58 years.

**Table 1:** Clinicopathologic characteristics of the LH and RH groups in the the overall cohort of Bismuth type IV PHC.

Variables	LH (n=146)	RH (n=72)	*P*
Age (years) *	58.00 (50.00-65.00)	56.50 (50.00-62.50)	0.340
Gender(Male/Female)	97/49	44/28	0.439
Total bilirubin at diagnosis (μmol/L) *	166.45 (48.90-277.50)	144.25 (42.40-284.40)	0.560
CA19-9 levels at operation (U/ml) *	292.90 (98.80-880.60)	191.90 (85.25-833.65)	0.448
Total bilirubin at operation (μmol/L) *	69.00 (39.40-107.60)	43.75 (29.80-83.50)	0.022
Preop. biliary drainage, n (%)			0.020
None	47 (32.19)	11 (15.28)	
Yes	99 (67.81)	61 (84.72)	
PTBD	89	55	
ENBD	5	6	
PTBD+ ENBD	4	0	
Other	1	0	
Portal vein embolization, n (%)	6 (4.11)	27 (37.50)	<.0001
Extent of hepatectomy, n (%)			0.181
Major	2	4	
Extended	144	68	
Tumor vascular invasion, n (%)			0.295
None	83 (56.85)	47 (65.28)	
Yes	63 (43.15)	25 (34.72)	
Portal vein, PV	25	14	
Hepatic artery, HA	14	6	
PV+ HA	24	5	
Postop. complications, n (%)			0.459
0/I/II	104 (71.2)	47 (65.3)	
IIIa/IIIb/ IV/V	42 (28.8)	25 (34.7)	
90-day mortality, n (%)	9 (6.16)	5 (6.94)	0.825
Resection margin, n (%)			0.783
R0	133 (91.10)	64 (88.89)	
R1/2	13 (8.90)	8 (11.1)	
Differentiation, n (%)			0.321
Well	1 (0.68)	1 (1.39)	
Moderate	140 (95.89)	70 (97.22)	
Poor	5 (3.42)	1 (1.39)	
Perineural invasion, n (%)	108 (73.97)	60 (83.33)	0.122
UICC TNM stage, n (%)			0.322
IIIB	109 (74.66)	49 (68.06)	
IIIC	33 (22.60)	21 (29.17)	
IV	4 (2.74)	2 (2.78)	

Values in parentheses are percentages unless indicated otherwise; *values are median (range); LH, left-sided hepatectomy; RH, right-sided hepatectomy; PTBD, percutaneous transhepatic biliary drainage; ENBD, endoscopic nasobiliary drainage; CA19-9, carbohydrate antigen 19-9; UICC, Union for International Cancer Control; t-test for means; Mann-Whitney test for medians; Chi-square or Fisher’s exact tests for categorical variables.

Left-side hepatectomy (LH) was performed for 146 patients (66.97%), while right-side hepatectomy (RH) was performed for 72 patients (33.03%). The distributions of age and gender were similar between the LH and RH groups. No difference was observed between the two groups in total bilirubin levels at diagnosis and CA19–9 levels at operation. But the total bilirubin level at the operation of the LH group tended to be higher than that of the RH group (p=0.022).

The demographics and characteristics of the 72 pairs of patients included in the matched cohort after PSM were summarized in **Supplementary Table S1**. The level of total bilirubin at operation was higher in the LH group compared to the RH in the matched cohort (p = 0.013).

### Preoperative management

None of the patients received neoadjuvant therapy prior to surgery. Preoperative biliary drainage was performed for most of the patients, and PTBD was the main method. The number of patients undertaken preoperative biliary drainage tended to be higher in the RH group than in the LH group of the overall cohort with a significant difference (p=0.020). And the patients in the RH group received portal vein embolization (PVE) prior to surgery more frequently to achieve a sufficient FLR (p<0.001) (**Table 1**). In the matched cohort, PVE was also performed more frequently in the RH group (p<0.001) (**Supplementary Table S1**).

### Surgical outcome and mortality

R0 resection was obtained in 90.37% (197/218) of patients of the overall cohort, while R1 was obtained in 9.17% (20/218) and R2 in 0.46% (1/218). The R0 rate was similar in both groups (91.18% (133/146) vs. 88.89% (64/72), p = 0.783). The extended hemihepatectomy, which was applied for most of the patients (212/218), was performed with no significant difference between the two groups (p=0.181). The other histopathologic findings in resected specimens, including tumor cell differentiation, perineural invasion, vascular invasion, and UICC stage, were not significantly different between the two groups either (**Table 1**).

Postoperative severe complications (grade 3, 4, and 5) occurred in 30.73% (67/218) of all patients. Although the rate of postoperative severe complications tended to be higher in the RH group, there was no statistical difference between the two groups (p = 0.459). A total of 14 patients in the overall cohort died within 90 days after surgery, including 9 patients in the LH group and 5 in the RH group. Liver failure is the leading cause of 90-day mortality (8/14), and biliary tract infection is the second leading cause (4/14). The 90-day mortality rate was similar in both groups (**Table 1**).

In the matched cohort after PSM, the rate of R0 resection, postoperative severe complications, and 90-day mortality was not significantly different between the two groups (**Supplementary Table S1**). And the other histopathologic variables, including tumor cell differentiation, perineural invasion, and lymph node status, were similar in both groups.

### Postoperative survival analysis

**Figure 2:**
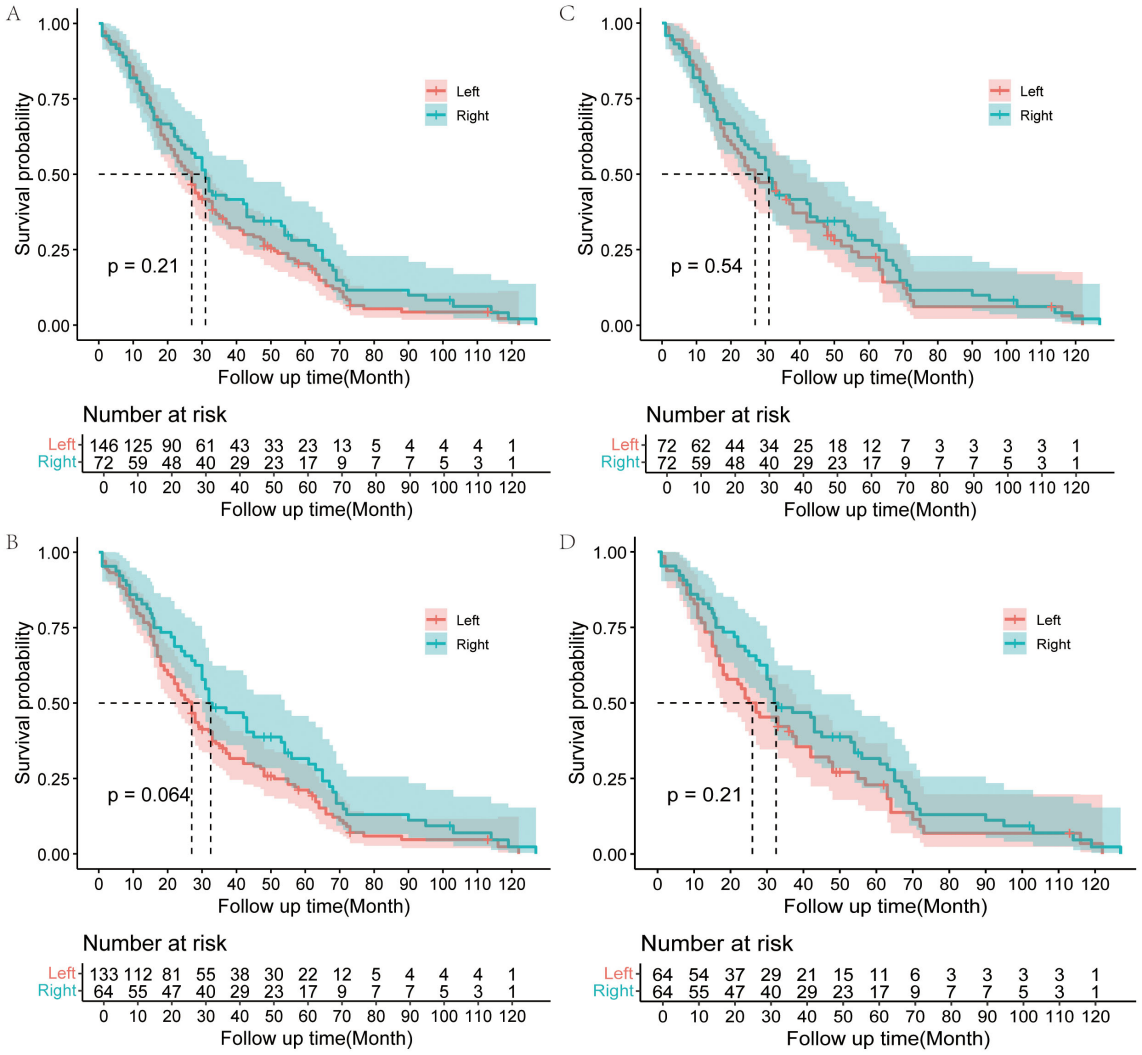
Kaplan–Meier analysis of OS for Bismuth type IV PHC patients. OS for Bismuth type IV PHC patients underwent LH or RH in the overall cohort **(A)**; OS for Bismuth type IV PHC patients underwent LH or RH with R0 resection in the overall cohort **(B)**; OS for Bismuth type IV PHC patients underwent LH or RH in the matched cohort after PSM **(C)**; OS for Bismuth type IV PHC patients underwent LH or RH with R0 resection in matched cohort **(D)**.

The overall survival rate for all 218 patients was 80.3% at 1 year, 37.6% at 3 years, and 22.9% at 5 years, with a median survival time (MST) of 27 months. And the respective 3- and 5-year survival rates were 35.2% and 20.4% in the LH group and 43.1% and 28.1% in the RH group (**Table 2**). The 3- and 5-year survival rate seems higher in the RH group, but Kaplan-Meier survival analysis shows no statistically significant difference between the two groups (p=0.21) (**Figure 2A**). However, survival for RH group patients who underwent R0 resection tended to be better than for LH group patients who underwent R0 resection, with borderline significance (p=0.064) (**Figure 2B**).

**Table 2:** Univariate and multivariate analyses of survival in patients who underwent LH or RH for Bismuth type IV PHC of the overall cohort.

Variables	n=	3-yr OS (%)	5-yr OS (%)	Univariate, *p*	Multivariate, *p*	HR	95% CI
Age,years							
≤58	113	45.6	30.8				
>58	105	29.5	14.2	0.002	0.003	1.56	1.16-2.10
Sex							
Female	77	33.7	21				
Male	141	40.1	23.9	0.325			
Total bilirubin at diagnosis (μmol/L)							
<326	179	40.6	25.6				
≥326	39	25.4	10.6	0.002	0.028	1.56	1.05-2.31
CA19-9 levels at operation (U/ml)							
≤328.6	121	49.3	31.3				
>328.6	97	23.6	12.7	<.0001	0.05	1.36	1-1.85
Total bilirubin at operation (μmol/L)							
<40	69	44.6	28.3				
≥40	149	34.7	20.5	0.007	0.23	1.56	1.05-2.31
Preop. biliary drainage							
Not performed	58	42.6	27.1				
Performed	160	36.1	21.4	0.067	0.9996	0.999	0.07-1.43
PVE							
Not performed	185	38.1	22.1				
Performed	33	36.4	26.9	0.91			
Side of hepatectomy							
Left	146	35.2	20.4				
Right	72	43.1	28.1	0.206			
Tumor vascular invasion							
No	130	47.4	29.2				
Yes	88	23.8	13.8	0.002	0.024	1.40	1.05-1.88
Resection margin							
R0	197	39.3	24.6				
R1/R2	21	23.8	6.35	0.021	0.104	1.48	0.92-2.38
Histology							
Well	2	50	50				
Moderate	210	37.9	22.9	0.82			
Poor	6	33.3	16.7	0.505			
N status							
N0	158	38.3	23.4				
N1-N2	60	36.7	22.2	0.92			
Perineural invasion							
No	50	36.8	23.6				
Yes	168	38	22.8	0.635			
Postop. complications							
0/I/II	151	45.4	25.8				
IIIa/IIIb/ IV/V	67	20.9	16.4	<.0001	<.0001	1.95	1.44-2.66

LH, left-sided hepatectomy; RH, right-sided hepatectomy; CA19-9, carbohydrate antigen 19-9; PVE, portal vein embolization; t-test for means; Mann-Whitney test for medians; Chi-square or Fisher’s exact tests for categorical variables.

In the matched cohort, postoperative factors were similar between the two groups. The median survival time was 30 months in the matched cohort. And the respective 3- and 5-year survival rates were 41.6% and 22.4% in the LH group and 43.1% and 28.1% in the RH group (**Supplementary Table S2**). The overall survival did not differ between the two groups in the matched cohort according to the Kaplan-Meier survival analysis (p=0.54), although the survival of the RH group seems better than that of the LH group (**Figure 2C**). There was also no significant difference in the survival between RH group and LH group patients who underwent R0 resection in the matched cohort (p=0.21) (**Figure 2D**).

### Univariate and multivariate analyses of survival of the Bismuth IV PHC patients who underwent LH or RH

To identify the predictors of long-term survival, univariable and multivariable analyses were performed on data from the 218 Bismuth type IV PHC patients of the overall cohort (**Table 2**) and 144 patients in the matched cohort (**Supplementary Table S2**). Univariable analysis of the overall cohort indicated that age, total bilirubin level at diagnosis, total bilirubin level at operation, CA19–9 levels at operation, preoperative biliary drainage, resection margin, tumor vascular invasion, and major postoperative complications were associated with survival. Further multivariable analysis identified age, CA19–9 levels at operation, tumor vascular invasion, and severe postoperative complications as independent prognostic factors (**Table 2**).

In the matched cohort, univariable analysis revealed that age, total bilirubin level at diagnosis, total bilirubin level at operation, CA19–9 levels at operation, preoperative biliary drainage, resection margin, tumor vascular invasion, and severe postoperative complications were prognostic factors, which was the same as in the overall cohort. But only age and severe postoperative complications were identified as independent prognostic factors in multivariable analysis (**Supplementary Table S2**).

## Discussion

Although there have been some previous studies on the comparison of left-sided resection and right-sided resection for perihilar cholangiocarcinoma, the conclusions of these studies have been contradictory and conflicting (7, 13, 21–25). Most of these studies incorporate all PHC patients receiving major liver resection regardless of the Bismuth-Corlette type (21, 22, 25), and others only compare trisectionectomy with hemihepatectomy (26–28). Although there have been studies that focus on only Bismuth type III PHC (24) or only Bismuth type IV PHC (29), the sample size included in the study was small. In the present study, we focus on comparing the results between the left-sided and right-sided hepatectomy for the Bismuth type IV PHC patients. And to the best of our knowledge, this is the first report in which the propensity score matching method was used to compare the outcomes in Bismuth type IV PHC patients who underwent LH or RH.

The general view is that the left-sided resection is more difficult and more likely to be combined with reconstruction of the branches of the hepatic artery or portal vein (30). In this regard, right-sided hepatectomy was more surgically advantageous than left-sided hepatectomy (5, 11). A previous study by Neuhaus P et al. showed that right-sided hepatectomy is superior to left-sided hepatectomy for PHC patients receiving liver resections regardless of Bismuth type (31). But in the present study, we found that left-sided resection was performed more frequently, accounting for 66.9% (146/218) of the overall cohort. From a functional viewpoint, the left-sided resection was less risky because the volume of the liver to be removed is small. And there was also less preparation before surgery for the left-sided resection, with a lower rate of portal vein embolization and preoperative biliary drainage. One study by Ebata et al. also showed that left-sided resection accounted for 71.8% of resections for Bismuth type IV tumors (32). And in another study including 50 PHC patients who underwent hepatectomy combined with arterial and portal vein resections, the left-sided resection was the main surgical strategy, resulting in 2% operative mortality and 30% 5-year survival (33). Moreover, this study also revealed that the left-sided resection accounted for 57.3% (209/365) of all resected patients (33). The above shows that left-sided resection was more widely performed, especially for Bismuth type IV PHC.

Nowadays, surgical resection with negative margins (R0) is still the only potentially curative method for the PHC patients. Although R1 resection could get better long-term survival compared with unresected PHC patients (5, 24, 32, 33), some studies have revealed that R0 resection was an independent prognostic factor influencing survival after surgical resection for PHC (7, 8, 27, 30). To achieve R0 resection, many surgeons have adopted an increasingly aggressive surgical approach to PHC such as trisectionectomy combined with vascular resection and reconstruction for Bismuth type III and IV tumors (26, 27, 29, 34). One study by Natsume et al. involved 201 PHC patients (Bismuth type I, II, IIIb, IV) who underwent left-sided hepatectomy revealed that trisectionectomy could result in a greater length of resected proximal bile duct, thus an increasing proportion of negative proximal ductal margin (35).

Hosokawa et al. reported that left trisectionectomy improved R0 resection rates compared with left-sided hepatectomy for PHC of the left-side predominance including Bismuth type II, IIIb and IV (34). While, in another study, the R0 resection was comparable between left trisectionectomy and left hemihepatectomy for Bismuth type III and IV tumors (7). Moreover, some other studies showed that survival did not differ between trisectionectomy and hemihepatectomy, despite different tumour loads (26–28, 35). And the incidence of severe complications seems to be high among PHC patients who underwent trisectionectomy (28, 35). In our department, we still applied the common hemihepatectomy-based approach mainly (36), which was different from the method of trisectionectomy. We also obtained a satisfactory R0 removal rate. Moreover, our series identified no significant differences in curability between LH and RH group.

As for the difference of the prognosis between LH and RH group, the conclusion of the present studies was contradictory. Some studies showed that RH could get better long-term survival due to the higher R0 resection rate (8, 37, 38). Some studies showed a favorable survival in the LH group (29). Nevertheless, other studies reveal that LH is comparable to RH in long-term survival (7, 13, 21, 22). But the previous studies have some limitations. Some of these studies included PHC patients receiving major liver resection regardless the differences in the malignancy degrees of different Bismuth-Corlette types. Some studies included relatively small sample sizes. In our research, we only focus on the survival of Bismuth type IV PHC patients after surgery. To date, literature and available data on outcomes of left-sided and right-sided resections for Bismuth type IV PHC patients were limited. One recent study focusing on Bismuth type IV PHC by Jeddou et al. showed that left trisectionectomies were associated with higher overall survival compared to right trisectionectomies (29), the conclusion of which was different from our study. In our study, no difference was observed in overall survival for the overall cohort after right-sided versus left-sided resections. Subgroup analysis showed a favorable survival for RH group patients who underwent R0 resection compared to LH group who underwent R0 resection, with borderline significance (p=0.064). To address the selection bias in a non-randomized design, the one-to-one propensity score matching (PSM) method was utilized. And the survival analysis between the LH and RH of the matched cohort (PSM) was not significantly different either. Therefore, this suggests that when planning the surgery, more comprehensive considerations should be given to the location of the tumor, the condition of the blood vessels, and the volume of the residual liver rather than which side of the liver to remove.

The comparison of the clinicopathological parameters between the LH and RH group of Bismuth type IV PHC patients showed that preoperative biliary drainage and portal vein embolization were performed more frequently in the RH group, and the total bilirubin level was lower in the RH group, which might contribute to the better survival of the RH group. Although the preoperative biliary drainage and portal vein embolization increased the waiting time for surgery and therefore increased the risk of tumor progression and metastasis, PTBD ± PVE was still necessary for Bismuth type IV PHC patients, especially for those with smaller FLR. Because these measures reduce the risk of postoperative liver failure, thus offset surgical risk.

Ratti et al. pointed that right-sided resections preserved a smaller liver remnant than corresponding left-sided resections, thus were correlated with higher mortality and morbidity rates, including the higher incidence of postoperative liver failure (8). However, the present study showed that the incidence of postoperative severe complications and the 90-day mortality rate were similar between the RH and LH groups, so that of the incidence of postoperative liver failure. This may be attributed to the meticulous perioperative management, including appropriate ENBD-based biliary drainage, portal vein embolization, early enteral feeding and intensive postoperative care.

In the previous studies, R0 resection and lymph node status have been reported as prognostic factors for PHC after surgery (7, 34, 39). But the present study revealed that R0 resection and lymph node status might not be the prognostic factors of OS for Bismuth type IV PHC after surgery, which was consistent with the study by Jeddou et al. (29). But in that study, R0 resection and lymph node status were independent prognostic factors of disease-free survival (DFS). We have found an interesting result that the variable of total bilirubin at operation loses significance after multivariate analyses, however the variable of total bilirubin at diagnosis does not. This might suggest that although the preoperative total bilirubin level can be reduced through PTBD or ENBD, the damage caused by cholestasis still leads to a poor prognosis. The severe postoperative complications were identified as an independent negative predictor of survival both in the overall cohort and matched cohort. This result indicated that surgical techniques should be refined to minimize the incidence of postoperative complications. Finally, since patients with older age, tumor vascular invasion, or higher CA19–9 levels at operation had significantly worse prognosis, postoperative adjuvant chemotherapy for such patients might be urgent.

We should acknowledge a few limitations in this comparative study. Firstly, this is a retrospective and single-center design study. Thus, the data of this study represent a single-center experience, which might be associated with a selection bias for the surgery related to the surgeon’s experience. Secondly, since many postoperative patients do not undergo follow-up and re-examination in our hospital, it is very difficult for us to record the exact time of tumor recurrence. Therefore, we are unable to compare the recurrence-free survival time of tumors between the two groups. Thus, a multi-center prospective randomized controlled trial should be conducted in the future to provide conclusive data. Last but not least we need to point out that, our department is a single high-volume HPB unit with advanced vascular resection capability, and the similar morbidity/mortality of the surgery for Bismuth type IV PHC may not be reproducible in smaller centers.

In conclusion, the present study showed that, compared to the LH for Bismuth type IV PHC, the postoperative morbidity rate and mortality rate of RH for Bismuth type IV PHC were comparable, although more meticulous perioperative management demanding. Although the right-sided hemihepatectomy for Bismuth type IV PHC patients, particularly for those who achieved R0 resection, showed a favorable survival from the Kaplan-Meier survival curve, no significant difference was observed in overall survival after right-sided versus left-sided resections for the overall cohort and the matched cohort after PSM.

## Data Availability

The raw data supporting the conclusions of this article will be made available by the authors, without undue reservation.
