# Supplementary material for: Right-side versus left-side hemihepatectomy for the treatment of Bismuth type IV perihilar cholangiocarcinoma: a comparative study
Source: Front Oncol. 2025 Nov 26;15:1663334. doi: 10.3389/fonc.2025.1663334 (PMC12689366; doi:10.3389/fonc.2025.1663334)
Supplement: Supplementary file 1 [file Table1.docx]

**TABLE S1** Clinicopathologic characteristics of the LH and RH groups in the matched cohort of the Bismuth type IV PHC

| **Variables** | **LH(n=72)** | **RH(n=72)** | ***P*** |
| --- | --- | --- | --- |
| **Factors used for propensity score matching** |  |  |  |
| Age (years) | 58.0 (58.0±10) | 56.4(56.4±8.71) | 0.309 |
| Gender(Male/Female) | 43/29 | 44/28 | 1.0 |
| Total bilirubin at diagnosis (μmol/L) * | 182 (84.7-281) | 144 (42.8-283) | 0.130 |
| Total bilirubin at operation (μmol/L) * | 78.5 (44.7-110) | 43.8 (29.8-82.4) | 0.013 |
| CA19-9 levels at operation (U/ml) * | 207 (59.5-438) | 192 (86.7-832) | 0.375 |
| Preop. biliary drainage, n (%) |  |  | 0.81 |
| No | 9(12.5) | 11 (15.3) |  |
| Yes | 63 (87.5) | 61 (84.7) |  |
| Portal vein embolization, n (%) |  |  | <0.001 |
| No | 66 (91.7) | 45 (62.5) |  |
| Yes | 6 (8.33) | 27 (37.5) |  |
|  |  |  |  |
| **Surgical outcome** |  |  |  |
| Postop. complications, n (%) |  |  | 0.199 |
| 0/I/II | 55 (76.4) | 47 (65.3) |  |
| IIIa/IIIb/ IV/V | 17 (23.6) | 25 (34.7) |  |
| 90-day mortality, n (%) | 4 (5.56) | 5 (6.94) | 1.0 |
| Resection margin, n (%) |  |  | 1.0 |
| R0 | 64 (88.9) | 64 (88.9) |  |
| R1/2 | 8 (11.1) | 8 (11.1) |  |
| Tumor vascular invasion, n (%) |  |  | 0.393 |
| No | 41 (56.9) | 47 (65.3) |  |
| Yes | 31 (43.1) | 25 (34.7) |  |
| Differentiation, n (%) |  |  | 1.0 |
| Well | 0 | 1(1.39) |  |
| Moderate | 70 (97.2) | 70 (97.2) |  |
| Poor | 2 (2.78) | 1(1.39) |  |
| Perineural invasion, n (%) |  |  | 0.669 |
| No | 15 (20.8) | 12 (16.7) |  |
| Yes | 57 (79.2) | 60 (83.3) |  |
| N status, n (%) |  |  | 0.782 |
| N0 | 52 (72.2) | 49 (68.1) |  |
| N1 | 19 (26.4) | 21 (29.2) |  |
| N2 | 1(1.39) | 2 (2.78) |  |

Values in parentheses are percentages unless indicated otherwise; *values are median (range); LH, left-sided hepatectomy; RH, right-sided hepatectomy; PTBD, percutaneous transhepatic biliary drainage; ENBD, endoscopic nasobiliary drainage; CA19-9, carbohydrate antigen 19-9; UICC, Union for International Cancer Control; t-test for means; Mann-Whitney test for medians; Chi-square or Fisher’s exact tests for categorical variables.
