# Supplementary material for: Right-side versus left-side hemihepatectomy for the treatment of Bismuth type IV perihilar cholangiocarcinoma: a comparative study
Source: Front Oncol. 2025 Nov 26;15:1663334. doi: 10.3389/fonc.2025.1663334 (PMC12689366; doi:10.3389/fonc.2025.1663334)
Supplement: Supplementary file 2 [file Table2.docx]

**TABLE S2** Univariate and multivariate analyses of survival in patients who underwent LH or RH for Bismuth type IV PHC of the matched cohort

| Variables | n= | 3-yr OS (%) | 5-yr OS (%) | Univariate, *p* | Multivariate, *p* | HR | 95% CI |
| --- | --- | --- | --- | --- | --- | --- | --- |
| Age，years |  |  |  |  |  |  |  |
| ≤58 | 75 | 48 | 33.7 |  |  |  |  |
| >58 | 69 | 36.2 | 15.4 | 0.006 | 0.028 | 1.506 | 1.05-2.17 |
| Sex |  |  |  |  |  |  |  |
| Female | 57 | 36.7 | 21.1 |  |  |  |  |
| Male | 87 | 46 | 27.8 | 0.2 |  |  |  |
| Total bilirubin at diagnosis (μmol/L) |  |  |  |  |  |  |  |
| <326 | 118 | 44.9 | 28.7 |  |  |  |  |
| ≥326 | 26 | 30.8 | 10.3 | 0.012 | 0.07 | 1.563 | 0.96-2.53 |
| CA19-9 levels at operation (U/ml) |  |  |  |  |  |  |  |
| ≤328.6 | 89 | 53.9 | 34.7 |  |  |  |  |
| >328.6 | 55 | 23.6 | 10.4 | 0.001 | 0.32 | 1.22 | 0.83-1.79 |
| Total bilirubin at operation (μmol/L) |  |  |  |  |  |  |  |
| <40 | 47 | 46.8 | 28.4 |  |  |  |  |
| ≥40 | 97 | 40.2 | 23.9 | 0.076 | 0.50 | 1.16 | 0.76-1.76 |
| Preop. biliary drainage |  |  |  |  |  |  |  |
| Not performed | 20 | 60 | 43.6 |  |  |  |  |
| Performed | 124 | 39.5 | 22.3 | 0.041 | 0.288 | 1.336 | 0.78-2.28 |
| PVE |  |  |  |  |  |  |  |
| Not performed | 111 | 44.1 | 24.7 |  |  |  |  |
| Performed | 33 | 36.4 | 26.9 | 0.784 |  |  |  |
| Side of hepatectomy |  |  |  |  |  |  |  |
| Left | 72 | 41.6 | 22.4 |  |  |  |  |
| Right | 72 | 43.1 | 28.1 | 0.054 |  |  |  |
| Tumor vascular invasion |  |  |  |  |  |  |  |
| No | 88 | 51.1 | 32.4 |  |  |  |  |
| Yes | 56 | 28.3 | 14.2 | 0.011 | 0.202 | 1.278 | 0.88-1.86 |
| Resection margin |  |  |  |  |  |  |  |
| R0 | 128 | 44.5 | 27.3 |  |  |  |  |
| R1/R2 | 16 | 25 | 8.33 | 0.053 | 0.113 | 1.574 | 0.90-2.76 |
| Histology |  |  |  |  |  |  |  |
| Well | 1 | 100 | 100 |  |  |  |  |
| Moderate | 140 | 41.4 | 24.6 | 0.419 |  |  |  |
| Poor | 3 | 66.7 | 33.3 | 0.399 |  |  |  |
| N status |  |  |  |  |  |  |  |
| N0 | 101 | 44.5 | 27.8 |  |  |  |  |
| N1-N2 | 43 | 37.2 | 19.9 | 0.486 |  |  |  |
| Perineural invasion |  |  |  |  |  |  |  |
| No | 27 | 48.1 | 31.7 |  |  |  |  |
| Yes | 117 | 41 | 24.2 | 0.62 |  |  |  |
| Postop. complications |  |  |  |  |  |  |  |
| 0/I/II | 102 | 50.9 | 28.9 |  |  |  |  |
| IIIa/IIIb/ IV/V | 42 | 21.4 | 16.7 | <.0001 | <.0001 | 2.025 | 1.37-2.99 |

LH, left-sided hepatectomy; RH, right-sided hepatectomy; CA19-9, carbohydrate antigen 19-9; PVE, portal vein embolization; t-test for means; Mann-Whitney test for medians; Chi-square or Fisher’s exact tests for categorical variables.
